# Evaluating the value and risks of endoscopic full-thickness resection for giant duodenal hamartomas

**DOI:** 10.1055/a-2780-0597

**Published:** 2026-02-03

**Authors:** Yanchen Lu, Lei Xu, Yuxuan Chen, Wenjun Wang, Xiaojie Hong, Chuantao Sun, Shuo Zhang

**Affiliations:** 1 587400 The Second Clinical Medical College of Zhejiang Chinese Medical University, Hangzhou, China; 2 Department of Gastroenterology, The Second Affiliated Hospital of Zhejiang Chinese Medical University, Hangzhou, China; 3 Key Laboratory of Traditional Chinese Medicine for the treatment of Intestine-Live of Zhejiang Province, Hangzhou, China

**Fig. 1:**
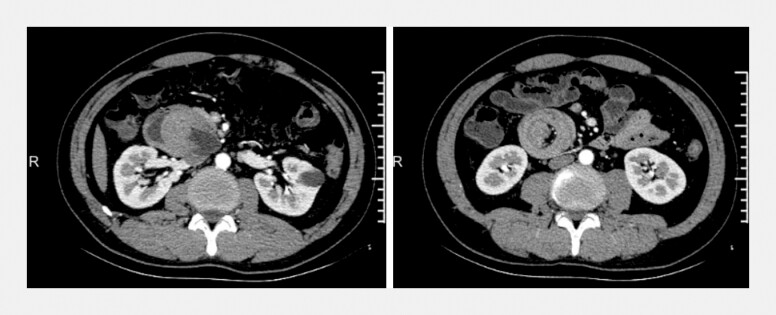
Contrast-enhanced abdominal CT showing a nodular soft tissue density in the descending duodenum, with partial jejunal intussusception into the duodenal lumen. CT, computed tomography.

**Fig. 2:**
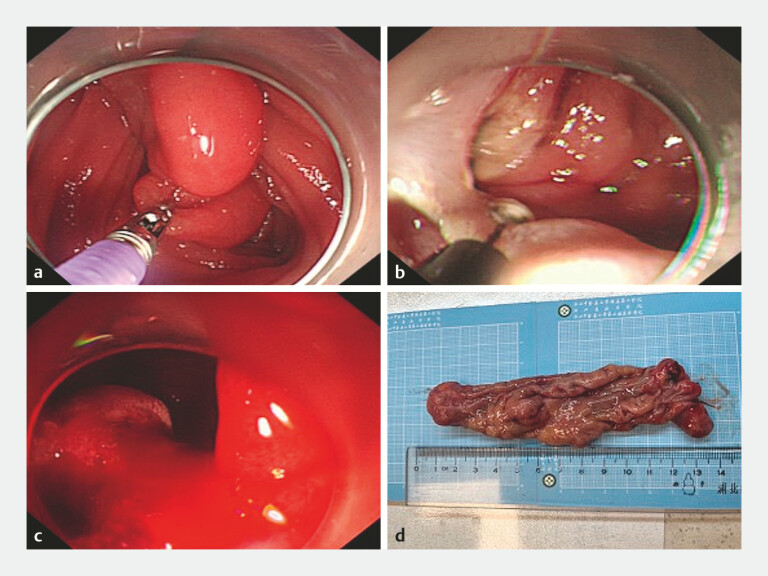
**a** Traction applied using biopsy forceps to reduce the intussusception of the giant duodenal mass. **b** Following marking of the lesion base, circumferential incision and layer-by-layer dissection were performed. Full-thickness resection was undertaken due to adhesion to the muscularis propria, achieving complete removal. **c** Sudden massive hemorrhage from the wound site obscured the visual field. Endoscopic hemostasis failed, necessitating surgical intervention. **d** Gross specimen of the endoscopic resected lesion, measuring approximately 14.0 cm × 4.0 cm.

**Fig. 3:**
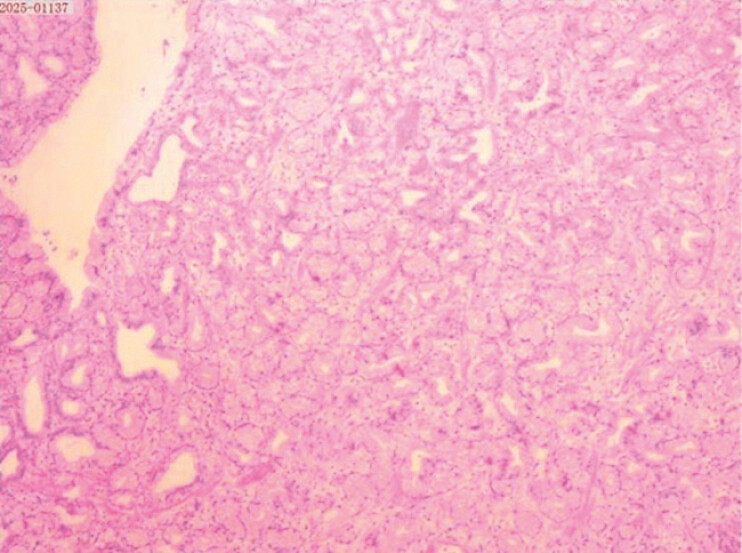
H&E staining (10 × 4) of the duodenal hamartoma. H&E, hematoxylin and eosin.

Case 1: A 43-year-old man presented with a 3-day history of abdominal distension. Contrast-enhanced computed tomography revealed a soft tissue mass in the duodenum accompanied by retrograde jejuno-duodenal intussusception ( Fig. 1 ). Gastroscopy identified a giant polypoid lesion. Following comprehensive preoperative evaluation and informed consent, the patient elected to endoscopic resection. During the procedure, a large mucosal protrusion was observed at the junction of the duodenal bulb and descending, prolapsing into a horizontal segment. After submucosal injection around the base of the lesion, a circumferential incision and layer-by-layer dissection were performed. Due to adhesions to muscularis propria, the lesion was completely resected via endoscopic full-thickness resection (EFTR) ( Fig. 2 ). However, sudden massive hemorrhage occurred from the wound, and endoscopic hemostasis attempts were unsuccessful ( Video 1 ). The patient was subsequently transferred for emergency surgical hemostasis, which was performed successfully. The patient recovered well postoperatively. Pathological examination confirmed the diagnosis of the hamartoma ( Fig. 3 ).

Two cases of giant duodenal hamartomas were completely removed through endoscopic full-thickness resection. However, the clinical outcomes were different.Video 1

**Fig. 4:**
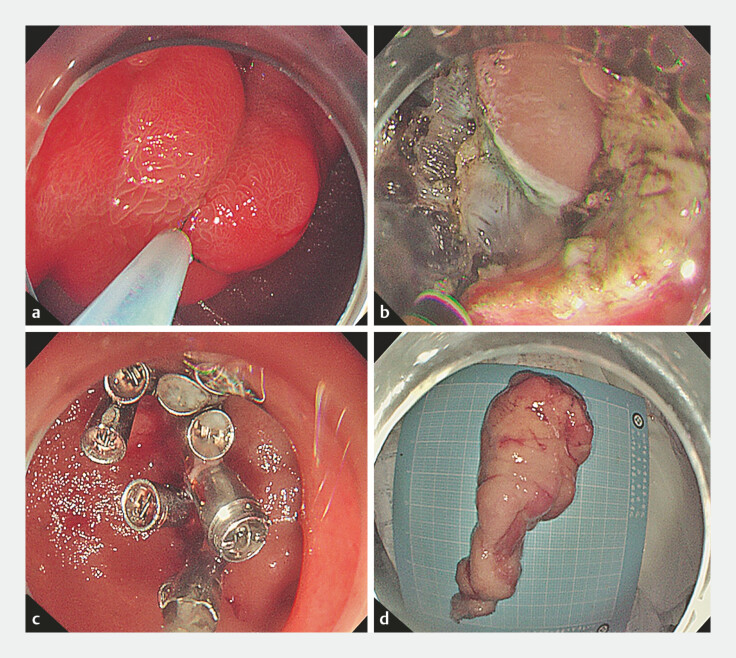
**a** A giant mass at the bulb-descending junction with a smooth surface, focally coarse mucosa, and erythematous, eroded mucosa at the head; the tumor prolapses into the descending duodenum. **b** Complete resection of the lesion via full-thickness resection. **c** Hemostatic forceps applied to vessel stumps at the wound site; the defect was closed using a combination of the through-the-scope twin clip and clips. **d** The gross specimen of the resected lesion, measuring approximately 9.0 cm × 3.5 cm.

**Fig. 5:**
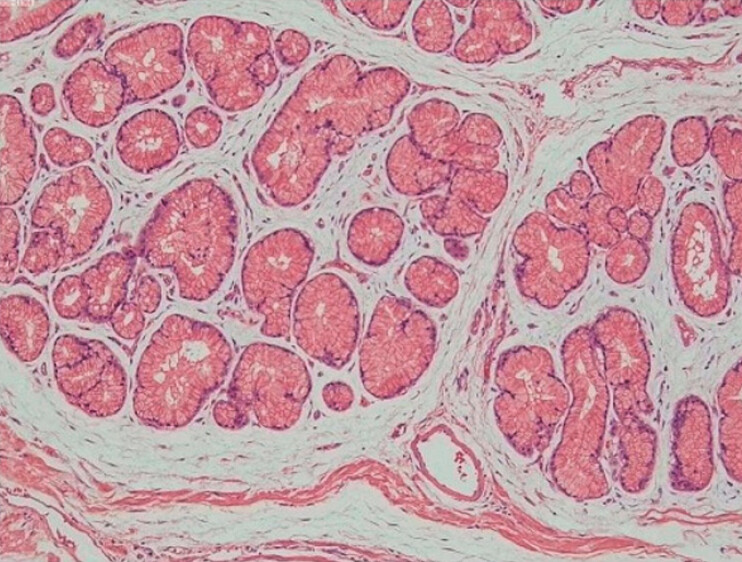
H&E staining (10 × 4) of the duodenal Brunner gland hamartoma. H&E, hematoxylin and eosin.

Case 2: A 38-year-old woman presented to a local hospital with a 2-week history of melena. Gastroscopy revealed an elongated mass in the descending duodenum with active bleeding at the tip, leading to refer to our institution. After thorough preoperative evaluation and informed consent, the patient opted for endoscopic treatment. Intraoperatively, the tumor was found to originate from the bulb-descending junction and prolapsed into descending. Following submucosal injection, circumferential incision and layer-by-layer dissection were performed, culminating in full-thickness resection at the base ( Fig. 4 , Video 1 ). The procedure was completed successfully, and the patient recovered well. Pathological analysis confirmed the diagnosis of Brunner’s gland hamartoma ( Fig. 5 ).

These cases illustrate that EFTR is an effective minimally invasive technique for achieving the complete resection of giant duodenal hamartomas. However, it is essential to recognize the potential risks of adverse events during endoscopic procedures. A careful balance between cutting and coagulation is critical for hemostasis, and timely surgical intervention should be readily available to ensure safety.

Endoscopy_UCTN_Code_TTT_1AO_2AG

